# Plasma-Stimulated Super-Hydrophilic Surface Finish of Polymers

**DOI:** 10.3390/polym12112498

**Published:** 2020-10-27

**Authors:** Miran Mozetič

**Affiliations:** Department of Surface Engineering, Jozef Stefan Institute, Jamova cesta 39, 1000 Ljubljana, Slovenia; miran.mozetic@guest.arnes.si

**Keywords:** plasma treatment, functionalization, tailoring surface properties, super-hydrophilicity, wettability, roughness

## Abstract

Super-hydrophilicity is a desired but rarely reported surface finish of polymer materials, so the methods for achieving such a property represent a great scientific and technological challenge. The methods reported by various authors are reviewed and discussed in this paper. The super-hydrophilic surface finish has been reported for polymers functionalized with oxygen-rich surface functional groups and of rich morphology on the sub-micrometer scale. The oxygen concentration as probed by X-ray photoelectron spectroscopy should be above 30 atomic % and the roughness as determined by atomic force microscopy over a few nm, although most authors reported the roughness was close to 100 nm. A simple one-step oxygen plasma treatment assures for super-hydrophilicity of few polymers only, but the technology enables such a surface finish of almost any fluorine-free polymer providing a capacitively coupled oxygen plasma that enables deposition of minute quantities of inorganic material is applied. More complex methods include deposition of at least one coating, followed by surface activation with oxygen plasma. Fluorinated polymers require treatment with plasma rich in hydrogen to achieve the super-hydrophilic surface finish. The stability upon aging depends largely on the technique used for super-hydrophilization.

## 1. Introduction

Products made from polymers or polymer composites are usually manufactured using extrusion or injection molding. The products will assume a rather smooth surface and the chemical nature of the polymer materials will enable moderate or poor wettability. Such a surface finish is often inadequate, in particular when the product should be coated with a third material, for example at gluing, printing, or deposition of a coating with specific functionality. The surface properties are modified by various techniques including treatment with aggressive chemicals, irradiation with beams of photons or charged particles, and mechanical treatments such as brushing or sandblasting. All these techniques enable modification of either the topology or the surface chemistry or both. Increased hydrophilicity is achieved by introducing polar functional groups on the polymer surface, and roughness is increased either by etching of the original material or deposition of a coating with rich morphology. The combination of the rich morphology on the sub-micrometer scale and polar surface functional groups will lead to a super-hydrophilic surface finish. Such a surface finish is probed by one of the simplest experimental techniques—deposition of a small droplet and measuring the contact angle between the substrate and the liquid. The super-hydrophilic surface finish will cause the spreading of a liquid of even high surface energy on a surface area much larger than the droplet diameter. The most commonly used liquid with high surface energy is distilled water. Furthermore, rough surfaces of super-hydrophilic materials favorite the capillary effect, so the water is distributed within the pores or channels over a large surface area. In any case, the water contact angle (WCA) is immeasurable low for polymer products of super-hydrophilic surface finish. The super-hydrophilicity is not as permanent, but the WCA slowly increases with storage time. The loss of hydrophilicity is usually called “hydrophobic recovery”.

A popular technique for modification of surface properties of various polymers is a brief treatment with non-equilibrium gaseous plasma. Different aspects of plasma–surface interaction have been elaborated on by various authors and summarized in review papers such as [1,2,3,4,5,6,7]. A polymer product is exposed to plasma which is a source of radiation (of particular importance is radiation in the ultraviolet (UV) and vacuum ultraviolet (VUV) range of photon energies), positively charged ions of different kinetic energies, and neutral reactive particles such as molecular fragments including neutral atoms. The surface finish depends on the treatment parameters. Different authors have used different plasmas, but only a few of them reported on the super-hydrophilic surface finish. This paper aimed to summarize recent achievements in this scientific niche and discuss the advantages and drawbacks of techniques that enable such a rarely-observed property of polymer materials.

## 2. State-of-the-Art

### 2.1. Exposure of Polymers to Low-Pressure Oxygen Plasma for Simultaneous Functionalization and Nanostructuring

The most straightforward technique for achieving the super-hydrophilic surface finish of polymers is direct exposure to a gaseous plasma of appropriate parameters. A classical work was reported by Tsougeni et al. [8]. They used a rather powerful low-pressure plasma reactor for modification of polymethyl methacrylate (PMMA) and polyetheretherketone (PEEK) discs. Plasma was sustained in pure oxygen at the pressure of 0.75 Pa and powered with an inductively coupled radio-frequency (RF) generator at the power of almost 2000 W. Additional biasing of the polymer samples was assured by another RF generator coupled capacitively. The electrodes for the capacitive discharge were made from aluminum. According to the authors, the experimental setup enabled the plasma-to-substrate voltage of 100 V. The polymer surfaces were therefore bombarded with positively charged oxygen ions of kinetic energy 100 eV. The sheath between the bulk plasma and the polymer substrates was collisionless because of the exceptionally low pressure. Plasma treatment time was varied for up to an hour. Both PMMA and PEEK samples assumed rich surface morphology on the sub-micrometer scale upon plasma treatment. Dense nanocolumns of height close to 100 nm appeared even after a minute of plasma treatment. This effect was explained by reactive ion etching of the polymers. The height of the nanocolumns increased almost linearly with the treatment time and reached 10 µm after half-an-hour treatment. Simultaneously, the concentration of aluminum (Al) in the surface film as probed by X-ray photoelectron spectroscopy (XPS) increased, too. The extremely rich surface morphology of both polymers after prolonged treatment was then explained by clustering the aluminum oxides on the sample surface, which caused the chemical etching by reactive ions highly inhomogeneous.

Samples of PMMA did not exhibit the super-hydrophilic surface finish for short treatment times when the Al concentration was marginal. Treatment time of an hour, however, enabled immeasurably low static water contact angle, which persisted for a few weeks after the treatment. The hydrophobic recovery was observed for prolonged aging, but the WCA remained below 70° even four months after the treatment. The results of Tsougeni et al. [8] are sound with other papers dealing with plasma hydrophilization of PMMA. Only moderate hydrophilicity of PMMA was observed upon oxygen-plasma treatment with the absence of surface contaminants likely to be formed by deposition of electrode materials because of sputtering [9]. Similar results were observed for PEEK, except that the hydrophobic recovery followed a different pathway: the samples treated in oxygen plasma for more than five minutes spontaneously assumed an almost super-hydrophobic property after a few months of aging. Namely, the WCA increased to about 150° after prolonged aging. The super-hydrophobic surface finish was also obtained after a brief plasma-enhanced deposition of Teflon-like film using the same plasma reactor but with C_4_F_8_ as the Teflon precursor. The authors found the technique scalable, but the long treatment times needed to obtain the super-hydrophilic surface finish may be a drawback.

Since the appropriate roughness was found crucial for super-hydrophilicity of polymers, Gogolides et al. further elaborated on the nanostructuring in the classical paper [10]. They proposed a couple of mechanisms responsible for the rich morphology of plasma-treated polymers: soft and hard etching inhibitors. The hard inhibitors are deposits originating from the walls of the discharge chamber or the electrodes, for example, metals, or metal oxides. The reactive oxygen species will not etch such hard inhibitors so the nano-areas on a polymer surface covered by inorganic material will remain fairly intact. Soft inhibitors are deposits that form on the polymer surface upon plasma treatment because of the condensation of etchable wall materials. The effect of reactor wall condition and reactor type on the roughness of polymeric surfaces were elaborated in [10]. The effects of both inhibitors were also predicted using the Monte Carlo simulation of the plasma-directed organization from ions and soft inhibitors by Vourdas et al. [11].

Kontziampasis et al. [12] treated thin films of poly(chloro-p-xylylene) (Parylene C) and studied the evolution of surface wettability, morphology, and functionalization using oxygen and argon plasmas sustained with inductively coupled RF discharge at the pressure of 1.33 Pa. The super-hydrophilic surface finish was observed only when treating the polymer with oxygen plasma for more than about 15 min. XPS showed oxygen concentration over 20 at.% even after a minute of plasma treatment. The O-concentration slowly increased thereafter and reached about 30 at.% when the super-hydrophilic finish as determined by WCA was observed. The roughness as deduced from atomic force microscopy (AFM) imaging was increasing almost linearly with the treatment time and reached about 170 nm when the super-hydrophilic effect was observed. Interestingly enough, the treatment with argon plasma in the same reactor caused a similar O-concentration, but the roughness was much smaller, so the water contact angle remained above 50° even after half-an-hour treatment. This paper, therefore, reveals the importance of the surface roughness on the hydrophilicity. They found the surface finish useful for the viability of cultured cardiomyocytes.

Recently, Kitsara et al. [13] disclosed a method for treatment of poly(vinylidene fluoride) (PVDF) scaffolds with a virtually permanent hydrophilic surface finish of scaffolds synthesized by electrospinning. As-fabricated scaffolds were treated with oxygen plasma produced in a reactor powered by a microwave generator. The discharge power was 200 W and the oxygen pressure 80 Pa. The super-hydrophilic finish was observed at the treatment time of 2 min. Slow hydrophobic recovery was observed, but the WCA remained at 35° even after two-years of storage. The as-deposited material was highly hydrophobic at WCA about 130°. The XPS showed about 7 at.% oxygen. The fluorine concentration remained at about 36 at.%. The original F concentration as deduced from XPS survey spectra was 45 at.%. The oxygen plasma treatment created some oxygen radicals and new moieties, mainly –CFO–CH_2_– and CF_2_–CHOR–, which were stable enough to prevent significant hydrophobic recovery. The surface finish was found useful for improving the cytocompatibility of Saos-2 cells.

Low-pressure capacitively coupled RF discharge was also employed by Perez-Roldan et al. [14] to obtain the super-hydrophilic finish of polyethylene terephthalate (PET). Helium or oxygen was used as source gases. The pressure in the processing chamber was as low as 0.033 Pa for the case of oxygen, and 1.3 Pa for helium. The discharge power was 75 W. The samples were placed on the grounded housing of the discharge chamber rather than on the electrode as in [8], so the ion kinetic energy was as low as about 10 eV. The super-hydrophilicity was observed in both gases after plasma treatment for about 10 min. Simultaneously with decreasing WCA, the surface roughness as deduced from atomic force microscopy (AFM) images increased. The immeasurably low WCA was determined at the roughness as low as about 2 nm. The oxygen concentration in the surface film as probed by XPS was increasing with treatment time and reached almost 40 at.% after half-an-hour treatment in helium plasma, less in the oxygen plasma. Complex evolution of different functional groups as deduced from high-resolution XPS C1s peaks was reported, and the hydrophobic recovery elaborated. Unlike Tsougeni et al. [8], no deposition of the electrode material was reported. The hydrophobic recovery occurred within a few hours.

An electrodeless RF discharge was used for PET modification by Vesel et al. [15]. Inductively coupled RF plasma was sustained in oxygen or nitrogen at the pressure of 75 Pa and discharge power of 200 W. Super-hydrophilic surface finish was observed only after treatment with oxygen plasma. For nitrogen plasma, the WCA remained at about 10°. The roughness that enabled super-hydrophilicity of oxygen-plasma treated samples was about 10 nm. The roughness of samples treated by nitrogen plasma as deduced from AFM images remained at about 4 nm. The nitrogen-treated PET samples assumed a high concentration of N as deduced from XPS survey spectra, while oxygen-treated samples were N-free. The concentration of oxygen on samples of super-hydrophilic finish was 44 at.%, i.e., similar to in [14]. Hydrophobic recovery upon storage at ambient conditions was observed within an hour after the plasma treatment. The de-convolution of high-resolution XPS C1s peaks revealed a rapid loss of the polar functional groups on the PET surface upon storage at ambient conditions.

Jaleh et al. [16] managed to obtain an almost super-hydrophilic surface finish of the polypropylene membrane upon exposure to oxygen plasma. The membranes were 0.2 mm thick with the 0.22 µm mean pore size. After cleaning the samples by chemical methods, the plasma treatment was performed in a low-pressure reactor powered with an RF glow discharge in capacitive mode. The discharge power was 25 W, the oxygen pressure 10 Pa, and the treatment time was varied between one and five minutes. The WCA decreased almost linearly with increasing treatment time and eventually fell below 20° after five-minutes of plasma treatment. The XPS revealed about 33 at.% oxygen in the surface film as probed by this technique. A well-defined sub-peak at 289 eV was observed from high-resolution C1s peak revealing the concentration of O–C=O functional group as large as 14%. The hydraulic conduction of plasma-treated membranes increased significantly because of the combined effect of etching and functionalization.

### 2.2. Atmospheric-Pressure Treatments

Several authors also probed atmospheric-pressure treatments for achieving super-hydrophilic surface finish. Recently, Gotoh et al. [17] reported on the super-hydrophilic surface finish of PET. They employed atmospheric pressure gaseous plasma jet (APPJ) for tailoring surface wettability. The PET substrate was treated with a nitrogen plasma of APPJ for less than 0.1 s. The oxygen concentration as probed by XPS increased for about 5 at.%, and 6 at.% of nitrogen was also found on the surface. The oxygen concentration that enabled the super-hydrophilic surface finish was 31 at.%. Similar wettability was also observed after treatment with UV radiation from a xenon excimer lamp of power 16 mW/cm^2^ for a minute, except that no nitrogen was found on the surface. The APPJ sustained in nitrogen is, therefore, a much more efficient way of super-hydrophilization of PET than deep UV radiation. A similar conclusion was recently brought also by Zhang et al. [18].

Numerous authors have treated PET by oxygen plasma at various experimental configurations but have not observed the super-hydrophilic effect. A recent review of the hydrophilicity of selected polymers has been published in [7]. The water contact angles reported by different authors are scattered significantly, so it is difficult to deduce any trends. The only clear conclusion brought in [7] is that aromatic polymers assume much lower water contact angles than aliphatic. The reasons are still unknown.

Several authors probed the wettability of porous polymer materials, but the super-hydrophilic finish for aliphatic polymers has rarely been reported. Asadinezhad et al. [19] managed to obtain various roughnesses on the polyvinyl chloride (PVC) from a few nm to several 100 nm and a high concentration of oxygen functional groups on the surface, but the nominal WCA obtained upon treatment with oxygen plasma was only 10°. Various discharge powers and treatment times were probed, but the super-hydrophilic surface finish was not achieved. The hydrophobic recovery, on the other hand, was rapid for this type of polymer.

An atmospheric-pressure dielectric barrier discharge (DBD) was employed by Ren et al. [20] for the treatment of polylactic acid (PLA) non-woven fabrics. The fabric was about 0.25 mm thick. The plasma treatment was performed in an argon atmosphere using a power supply operating at the frequency of 20 kHz and voltage 17 kV. The fibers were smooth at the average roughness deduced from AMF imaging as small as 2 nm and became rougher upon the plasma treatment. The roughness increased with treatment time and became 6 nm after about 100 s of plasma treatment. The WCA decreased monotonously with the treatment time. The original WCA was about 122°. Half-a-minute treatment caused a WCA of 50°, and the super-hydrophilic surface finish was observed after about 90 s of plasma treatment. The surface wettability remained stable for a few months. The oxygen concentration as probed by XPS increased from the original 38 to 42 at.% and traces of nitrogen were also detected.

Mahdieh et al. [21] used a corona discharge in ambient air at atmospheric pressure to tailor surface properties of polyester/cellulose fabrics. The textile samples were carefully cleaned before the plasma treatment. They managed to sustain the discharge at a voltage as low as 100 V. The discharge at rather low power caused increased surface morphology on the nano-scale, while powerful discharge also changed morphology on the µm scale. The significant etching was observed by weighting the fabrics before and after the plasma treatment. The samples were probed by Attenuated total reflectance Fourier transform infrared spectroscopy (ATR-FTIR), and the authors found cyanide groups on the plasma-treated samples. A moderate discharge power caused the super-hydrophobic properties of the treated samples, while the discharge power of 800 W resulted in a super-hydrophilic surface finish. The average roughness for both the untreated and plasma-treated samples as probed by AFM was around 200 nm. Hydrophobic recovery was observed for super-hydrophilic surface finish.

Akkan et al. [22] treated medical-grade PEEK foils of thickness of 1 mm to obtain a super-hydrophilic surface finish. The foils were treated with a plasma sustained in a mixture of argon and oxygen (3:1) by capacitively coupled RF discharge. The discharge power was estimated to about 270 W. Pulsed laser beams were employed to drill small periodical holes or a radius of about 10 µm. The plasma treatment time was as long as one hour. Such a long treatment caused significant modifications of PEEK surface properties and formation of nanofibrous structures, similar to what has been observed by Tsougeni et al. [8]. No electrode material was observed on the polymer surface, though. The structures, in combination with the oxygen-rich functional groups, enabled super-hydrophilic surface finish. Treatment by laser beams resulted in the super-hydrophobic properties even though a rather large concentration of oxygen functional groups as determined from XPS spectra persisted after the laser treatment. The authors found the technique scalable and promising for any application where the wettability of polymer surfaces is desired.

### 2.3. Complex Procedures

An alternative to one-step plasma treatment to obtain the super-hydrophilic surface finish is an activation of the polymer substrate and deposition of a thin film with rich morphology, followed by additional plasma treatment. Such an approach was adopted by several authors, and the selected papers are briefly described below.

Kuzminova et al. [23] deposited hydrogenated carbon nanoparticles of spherical shape and average diameter of about 110 nm onto smooth substrates. The deposition was performed using a plasma reactor filled with a mixture of argon and hexane. The reactor was equipped with an RF generator of power 120 W. The generator was coupled with plasma in the capacitive mode. The hexane was radicalized because of plasma conditions, and the radicals caused the formation of spherical nanoparticles in the gas phase. The nanoparticles deposited on the substrate surface and formed a coating of rich morphology. In the next step, a super-hydrophilic coating was deposited using a capacitively coupled RF plasma in a mixture of hexamethyl disiloxane (HMDSO) and oxygen. A layer consisting of oxygen, silicon, and carbon was deposited on the surface on nanoparticles and probed by WCA. The water contact angle was immeasurably low.

In another paper by the same group, Kylian et al. [24] used a similar technique as Kuzminova et al. [23] but studied the formation and stability of the super-hydrophilic surface finish thoroughly. Kylian et al. [24] selected polyethylene naphthalate (PEN) and polymethyl methacrylate (PMMA), with foils of thickness of 50 µm as substrates. The as-received foils exhibited different morphologies. The foils were coated with silver nanoparticles using the same equipment as Kuzminova et al. [23], but the deposition of Ag nanoparticles was accomplished using a direct current (DC) magnetron sputtering device as a source of Ag atoms. The agglomeration of Ag to nanoparticles occurred in the gas phase, and the nanoparticles covered the polymer substrates. The nanoparticles caused a coating of practically the same morphology on both polymers. The final deposition technique was coating with a thin film containing Si, O, and C using a conventional plasma deposition from HMDSO precursor in the presence of oxygen. The roughness, as determined by AFM, was about 80 nm. A small concentration of Ag nanoparticles and thus smaller roughness did not lead to a super-hydrophilic surface finish. The samples were aged for as long as two years, and the super-hydrophilic finish remained practically intact. The same materials with the same coating but without the film of Ag nanoparticles exhibited slow hydrophobic recovery and lost the super-hydrophilic properties several days after the deposition.

Lin et al. [25] prepared a complex membrane consisting of polydimethyl siloxane (PDMSO), polyvinyl alcohol (PVA), and poly(2-methacryloyloxyethyl) phosphorylcholine (PMPC) layers. The membrane was deposited onto the shark-skin template. The PMDSO was first deposited onto the template and treated by oxygen plasma using an atmospheric-pressure plasma jet. The plasma-treated PMDSO membrane was coated with PVA by spin-coating and dried in a vacuum oven. Finally, the methacryloyloxyethyl phosphorylcholine (MPC) monomer was coated and exposed to an oxygen plasma to form a brush-like structure. The as-prepared membrane exhibited a super-hydrophilic effect on the PMPC-coated side, and moderately hydrophobic with the WCA of about 120° on the PDMSO side. The material was found useful for wound dressings since the hydrophilic side enabled optimal sorption of exudate, and the opposite (hydrophobic) side prevented water penetration. Furthermore, the hydrophobic side exhibited anti-bacterial properties.

Another technique for achieving a super-hydrophilic surface finish of PDMSO foils was elaborated by Ruben et al. [26]. Microchannels of width and depth about 200 µm were created on a surface of the foil by replica molding. The samples were exposed to oxygen plasma at a pressure between 10 and 40 Pa. Plasma was sustained with a capacitively coupled RF generator at the power as low as 15 W. The WCA was decreasing with increasing pressure for constant treatment times and the super-hydrophilic surface finish was observed only at the largest pressure of 40 Pa. The authors concluded that the surface finish was a result of the interaction of the PDMSO foil with the O-atoms. This conclusion was based on the measurements of relative intensities of the O-atom emission line at 777 nm and O_2_^+^ radiation at 563 nm. The plasma-activated microchannels enabled spontaneous capillary flow, which was found useful in such microdevices.

Treatment of plasma-deposited PMDSO-like films with oxygen plasma was also elaborated by Wei et al. [27]. Plasma polymerization from HMDSO precursor was performed at the pressure of 10 Pa and RF power of 200 W for 600 s to obtain films of thickness about 100 nm. Oxygen plasma treatment was performed at 1.8 Pa, 10 W, and various treatment times. The super-hydrophilic surface finish was obtained after a minute of oxygen-plasma treatment. No significant etching was observed, but the surface of the PMDSO-like film assumed a high concentration of oxygen—about 57 at.%. Spontaneous hydrophobic recovery was observed upon storing at ambient conditions, but storage in distilled water suppressed this effect significantly. The surface roughness, as determined by AFM, remained practically intact after the oxygen plasma treatment. The surface finish was found useful for spreading the fibroblast cells, although their proliferation was not affected much. Similar results were reported by the same group in another paper [28].

Airoudj et al. [29] also managed to create textiles with highly hydrophobic properties on one side and super-hydrophilic finish on the other. The latter effect was achieved by the treatment of cotton textiles in plasma of maleic anhydride (MA) vapor at the pressure of 20 Pa. Inductively coupled RF plasma in the E-mode was sustained for 300 s at the discharge power of 30 W. The thickness of the polymer film deposited at these conditions was about 40 nm. The polymer film was hydrolyzed after the plasma treatment using a water solution of HCl at pH 4 for 120 s. The hydrolysis enabled the formation of highly polar O=C-OH groups on the textile surface and thus immeasurable low contact angles for both water and hexadecane. The concentration of oxygen in the surface films of super-hydrophilic coating (25 at.%) as probed by XPS was much lower than in pristine cotton textile (33 at.%) or theoretical composition of MA (43 at.%). Good durability of such materials was observed even after several washing cycles.

While the surface functionalization of fluorine-free polymers by oxygen plasma treatment always causes increased wettability (rarely the super-hydrophilic surface finish, though), the activation of Teflon or similar polymers remains a scientific challenge. The only report on the immeasurable low WCA was published recently by the group from Saitama, Japan. Nguyen and Yajima [30] used low-pressure plasma sustained by an inductively coupled RF discharge for tailoring surface properties of polytetrafluoroethylene (PTFE) foils. The experimental setup was similar to that employed by Vesel et al. [15]. A variety of gases or gas mixtures were probed, but the best results in terms of the super-hydrophilic surface finish were observed when using a mixture of argon, ammonia, and water vapor. The discharge power was about 100 W. According to the authors [30], the plasma sustained in such a gas mixture is rich in NH_3_^+^ ions which are key species involved in the de-fluorination of the PTFE surface film. The NH_3_F^+^ ions are created on the PFTE surface what Nguyen and Yajima [30] found beneficial for the de-fluorination of the PTFE surface. Furthermore, the combined effect of bond-scission by UV absorption and OH radicals caused a significant concentration of oxygen-rich functional groups on the surface of plasma-treated samples. As many as 27 hydroxyl groups per 100 carbon atoms were reported when the super-hydrophilic surface finish was achieved. The chemistry involved upon the de-fluorination of PTFE using hydrogen-containing plasma of extensive VUV radiation was recently elaborated by Lojen et al. [31].

The results reported for the plasma treatment of polymers by the above-cited authors are summarized in Table 1. The column “AFM” of Table 1 indicates that the super-hydrophilicity was observed at various roughnesses as calculated from AFM images. For example, Perez-Roldan et al. [14] reported such a wettability of PET for the roughness as low as 2 nm, while Tsougeni et al. [8] haven’t observed an immeasurable low WCA before the roughness of 100 nm has been achieved. Some authors even reported larger values, but always below a micrometer. From these observations, one can only conclude that the necessary condition is roughness in the sub-micrometer scale and the effect depends on other parameters, i.e., peculiarities of treated materials and treatment methods. A large oxygen concentration in the surface film is a necessary condition as revealed from column “XPS”. Most authors obtained such a large concentration by etching the surface film of the polymer product and functionalization with oxygen groups. The minimal oxygen concentration for observing the super-hydrophilicity of 25 at.% was reported by Airoudj et al. [29]. The results might have been misinterpreted since the O-concentration was below the value typical for untreated polymers used in that study. All other authors reported O-concentration above 30 at.%. Here, it is worth to stress that the technique used for the determination of oxygen concentration (XPS) does not probe the surface atoms only, but rather gives the composition averaged over the escape depth of photoelectrons (several nm for most polymers).

## 3. Summary and Discussions

The literature survey indicates a couple of methods for plasma-stimulated super-hydrophilicity of polymer materials:

1. one-step exposure to reactive plasma, typically oxygen;
2. two- or several-steps involving at least one deposition of a coating with high roughness followed by surface activation with oxygen plasma.

### 3.1. One-Step Exposure to Oxygen Plasma

**Figure 1:**
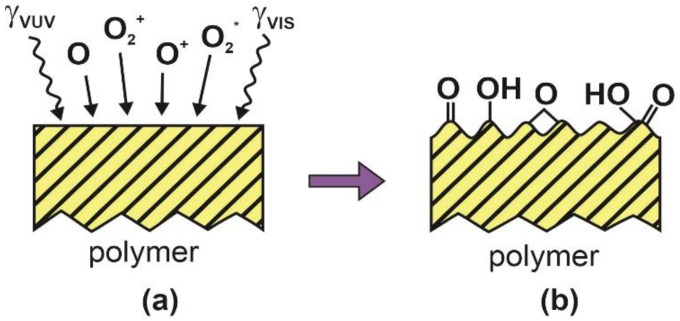
(**a**) Originally smooth polymer. (**b**) Polymer after exposed to oxygen plasma assumes rich morphology and becomes functionalized with polar surface groups.

The treatment of polymers with oxygen-containing plasma is a natural choice for surface hydrophilization since the oxygen-rich functional groups assure for high wettability of a polymer material. The super-hydrophilic surface finish, however, is achieved only by providing the plasma treatment also causes an appropriate roughness. Schematic of the one-step procedure for achieving super-hydrophilic surface finish is presented in Figure 1. A polymer sample is subjected to radiation arising from transitions between O-atoms, which appear predominantly in the red and VUV part of the spectrum. The sample is also exposed to neutral atoms in the ground and metastable excited states, positively charged molecular and atomic ions, and metastable molecules (Figure 1a). The VUV radiation causes bond scission in the surface film [18], while the atomic and molecular species may interact chemically, causing both functionalization and etching. Exposure to reactive oxygen plasma species rarely leads to the super-hydrophilic surface finish of polymers. The reason is an inappropriate combination of roughness and functionalization. Although not often mentioned in scientific literature, the exposure to non-equilibrium oxygen plasma affects not only the very surface but also a thicker surface film. For example, Bruce et al. [32] exposed polystyrene to energetic ions and explained the evolution of the surface roughness by buckling instability. The surface layer became stiff upon ion treatment, and a large mismatch in the stiffness of the affected layer and bulk polymer wrinkles the surface layer to minimize the elastic energy as shown schematically in Figure 1b. The explanation provided by Bruce et al. [32] takes into account a rather large kinetic energy of positively charged ions. In many cases, however, the polymer samples assume the floating potential, so the kinetic energy of ions is too low to trigger such an effect. The stiffness of the surface film may also be changed by other effects, for example, synergetic effects of VUV radiation and exothermic surface reactions, as explained by Lehocky et al. [33]. Another explanation for rich surface morphology upon exposure of polymer samples to oxygen plasma is the preferential etching of the amorphous phase as elaborated by Junkar et al. [34]. In any case, only a handful of polymers become super-hydrophilic upon exposure to oxygen plasma as in Figure 1. Furthermore, the super-hydrophilic effect using the technique of Figure 1 is far from being permanent as a rapid hydrophobic recovery was reported by all authors using this technique. Fluorinated polymers will not become super-hydrophilic using this technique since the reactive oxygen species from plasma will not form polar oxygen-rich surface functional groups but will instead cause etching only. Such polymers are better treated according to the technique described by Nguyen and Yajima [30].

**Figure 2:**
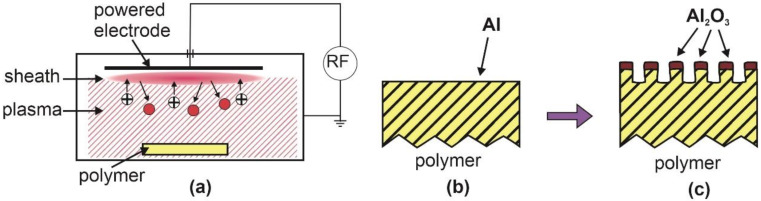
(**a**) Schematic of capacitively coupled radio-frequency (RF) plasma reactor. (**b**) Species impinging the polymer surface, and (**c**) resultant surface finish.

The hydrophobic recovery was suppressed using a capacitively coupled RF discharge. The experimental setup is shown schematically in Figure 2. The powered electrode is much smaller than the grounded housing, so a strong DC self-biasing occurs [35]. A sheath forms spontaneously next to the powered electrode at such experimental conditions. The ions entering the sheath from bulk plasma are accelerated in the high electric field within the sheath and gain a rather large kinetic energy. They bombard the powered electrode and cause weak sputtering of the electrode material. The atoms sputtered from the powered electrode condensate on the polymer substrate, as shown in Figure 2b. A thin film of the electrode material (often aluminum) is deposited on the polymer sample. If the polymer itself is biased (for example, by placing a piece of polymer on the powered electrode), the sample will be subjected to energetic oxygen ions causing etching of the organic compound. The electrode material will form clusters that will prevent chemical etching on the top so that etching will occur only within gaps between the inorganic clusters. The final effect will be nanostructuring, as shown in Figure 2c. The clusters of oxidized electrode material will persist on the top of the polymer sample and assure a long-term super-hydrophilic surface finish, as elaborated by Gogolides’ group [8]. The surface finish depends predominantly on the properties of inorganic clusters rather than the polymer substrate, so this technique applies to practically any polymer. The adhesion between the polymer substrate and the inorganic clusters may not always be optimal. Another drawback may be excessive heating of the polymer sample upon bombardment with the positive ions. Fluorinated polymers may become super-hydrophilic using this technique, but the adhesion of the inorganic clusters is often inadequate.

### 3.2. Deposition of a Coating with High Roughness Followed by Surface Activation

**Figure 3:**
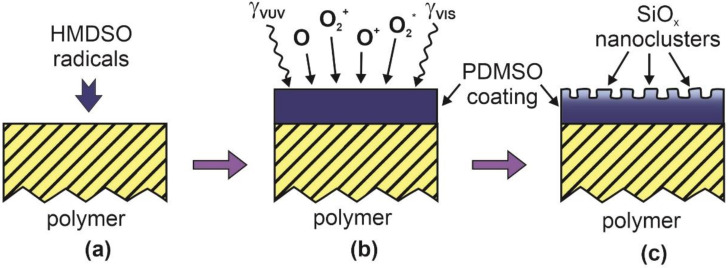
Schematic of a method for hydrophilization of polymers by deposition of a thin polydimethyl siloxane (PDMSO)-like coating followed by oxygen plasma treatment. (**a**) Polymer exposure to hexamethyl disiloxane (HMDSO) radicals to deposit PDMSO-like coating, (**b**) treatment of PDMSO-like coating in O_2_ plasma, and (**c**) etching of organic part of PDMSO and formation of nanostructured SiO_2_-like surface.

The drawbacks of the one-step technique are irrelevant when a two-step procedure is employed to obtain the super-hydrophilic surface finish of various polymers. The polymer material is first activated by a brief treatment in plasma of virtually any gases. The activation will assure for a certain concentration of polar functional groups that will, in turn, assure for good adhesion of a uniform coating. According to the state-of-the-art, a PDMSO-like coating is preferred. A widely used method for deposition of such coatings is an application of weakly ionized plasma sustained in HMDSO. The gaseous precursor partially dissociates upon plasma conditions, and the radicals stick to the surface of any material facing plasma and form a thin film containing carbon, hydrogen, silicon, and oxygen. The concentrations of these elements in the deposited film depend enormously on the experimental conditions [36]. The sample is then exposed to oxygen plasma to etch the organic component from the surface of the PDMSO-like coating leaving dense nanoparticles containing silicon oxides. A schematic of the procedure is shown in Figure 3.

Prior to the deposition, the surface of the polymer substrate should be free from impurities and preferably activated by brief plasma treatment. Such treatment is used routinely on an industrial scale [37]. Plasma could be sustained at a very low power density in different gases, including the residual atmosphere in the plasma reactor [38]. Namely, even weak functionalization of a fluorine-free polymer with polar functional groups will assure the desired adhesion of a thin film synthesized by plasma polymerization. The polymer samples are exposed to a variety of radicals presented in weakly ionized HMDSO plasma, as shown in Figure 3a. Prolonged treatment (typically a few minutes) will enable the deposition of a PDMSO-like coating without affecting the properties of the substrate. Once the film of an appropriate thickness (often around 100 nm) is deposited, the samples are treated by oxygen plasma (Figure 3b). The reactive oxygen species will etch away the organic component of the PDMSO-like coating, thus forming densely distributed nanoparticles consisting predominantly of silicon oxides. The as-synthesized nanoparticles assure for the super-hydrophilic surface finish. This technique was used by Ruben et al. [26] and Wei et al. [27]. Both authors reported super-hydrophilic surface finish and the low concentration of carbon as deduced from XPS spectra. The nanoparticles were probably almost free from carbon since the C signal probably arose from photoelectrons emitted from the less-affected segment of the coating between the neighboring nanoparticles. Even a very mild plasma treatment is sufficient for obtaining the surface finish as in Figure 3c since both authors used a discharge power of solely 15 and 10 W, respectively. The technique is especially useful for specific applications (capillary flow and proliferation of biological cells). However, the adhesion of any coating on the substrate of the surface finish as in Figure 3c may be questionable. A similar, but more complex technique as in Figure 3 was also adopted by Lin et al. [25] and Airoudj et al. [29].

**Figure 4:**
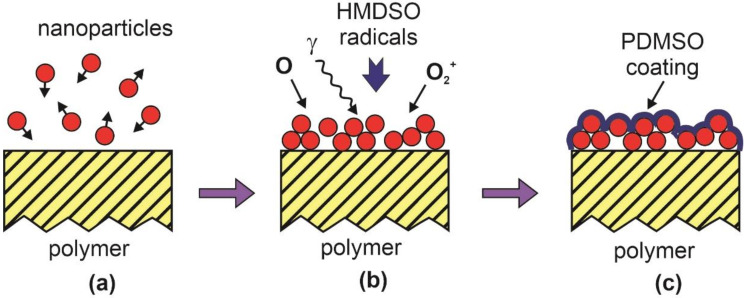
Schematic of a method for hydrophilization of polymers by deposition of a thin coating of nanoparticles followed by deposition of carbon-depleted PDMSO-like coating. (**a**) Deposition of nanoparticles, (**b**) treatment of a layer of deposited nanoparticles in O_2_ plasma with admixed HMDSO, and (**c**) formation of PDMSO coating on the layer of nanoparticles.

The method presented schematically in Figure 3 could be simplified by merging the deposition and oxygen plasma treatments, for example by deposition of SiO_x_ nanoparticles from gaseous plasma sustained in oxygen with an admixture of HMDSO. The nanoparticles prepared this way, however, may not adhere to the polymer surface. This drawback is suppressed by depositing nanoparticles of a third material onto a plasma-activated polymer substrate. The technique was elaborated by Kuzminova et al. [23] and Kylian et al. [24]. The schematic of such a procedure is shown in Figure 4. A polymer substrate is first activated similarly as in Figure 3. Then, and without breaking vacuum conditions, the substrate is exposed to nanoparticles of the typical diameter of 100 nm, as shown in Figure 4a. The nanoparticles form a thin layer on the polymer substrate (Figure 4b). The layer of nanoparticles is then coated with a thin film of carbon-depleted PDMSO-like coating (Figure 4c). The coating is rich in oxygen and thus provides binding sites for any further coating. The technique is scalable and assures for long-terming super-hydrophilicity. The adhesion between the nanoparticles and the polymer substrate may limit the application of this method.

## 4. Conclusions

Plasma methods for obtaining super-hydrophilic surface finish of polymer materials were reviewed and discussed. The straightforward technique is a treatment with oxygen plasma. The combination of oxygen-rich surface functional groups and roughness on the sub-micrometer scale enables super-hydrophilicity of any polymer providing the morphology and functionalization are adequate. So far, this technique was elaborated only for Poly(ethylene terephthalate) samples. A rather broad range of roughness as deduced from AFM imaging was reported to assure the super-hydrophilic surface finish. The effect, however, is of short duration since the hydrophobic recovery was reported by all authors. Typically, the superior effects are lost already several minutes after the plasma treatment. This technique is therefore useful only in cases when such a surface finish is needed for immediate deposition of a third coating. For example, before printing, gluing, or painting. The adhesion of any coating on a polymer treated using this method should be particularly good—any liquid (paint, glue, etc.) will enter the pores and interact chemically with the surface functional groups.

A more sophisticated technique is a simultaneous etching of a polymer material and depositing an inorganic material. A feasible configuration is a plasma reactor that enables the deposition of small quantities of a reactive metal (such as aluminum) upon the treatment of a polymer sample with oxygen plasma. The technique was elaborated for few polymers but should apply to any fluorine-free polymer. Namely, the super-hydrophilicity using this method is due to the presence of clusters of metal oxides on the surface of extremely rough polymers. The adhesion of any coating should be equally good as in the case of metal-free surface finish, but a possible drawback of this technique is long treatment time.

An alternative to such one-step methods is a deposition of various coatings of rich morphology followed by surface activation with oxygen plasma. Such treatments apply to any polymers, but the adhesion may be problematic since the coatings consist of nanoparticles that may not stick well either to the polymer substrate or any coating deposited after obtaining the super-hydrophilic effect.

## Figures and Tables

**Table 1:** Summary of the recent papers reporting super-hydrophilic surface finish of polymers.

Author	Material	Discharge, Power (W)	Gasses	Pressure (Pa)	Deposition (D)/Etching (E)	XPS			AFM
						C	O	Other	
[8]	PMMA disc	RF, 2000	O_2_	0.75	E	40	38	Al	>100 nm
[8]	PEEK disc	RF, 2000	O_2_	0.75	E	44	34	Al	>100 nm
[22]	PEEK foil	RF, 270	Ar–O_2_	N/A	E	57	43		SEM nanofibrils
[12]	ParyleneC film	RF, 1000	O_2_	1.33	E	67	30	Cl	170 nm
[13]	PVDF electrospun	MW, 200	O_2_	80	E	57	7	F	N/A
[14]	PET foil	RF, 75	O_2_ or He	0.033 or 1.3	E	70	30		2 nm
[15]	PET foil	RF, 200	O_2_	75	E	56	44		10 nm
[17]	PET foil	APPJ	N_2_	10^5^	E	63	31	N	N/A
[20]	PLA non-woven	HF	Ar	10^5^	E	57	42	N	6 nm
[16]	PP membrane	RF, 25	O_2_	10	E	67	33		SEM
[21]	Polyester/cellulose fabrics	DC	air	10^5^	E	N/A	N/A		200 nm
[30]	PTFE foil	RF, 100	Ar, NH_3_, H_2_O	30	E	N/A	N/A		N/A
[23]	Coated C:H nanoparticles	RF, 40	HMDSO, O_2_	4	D	5	60	Si	110 nm
[24]	Coated Ag nanoparticles	RF, 40	HMDSO, O_2_	4	D	5	58	Si	80 nm
[26]	PDMSO	RF, 15	O_2_	40	E	25	41	Si	SEM microchannels
[27]	HMDSO film	RF, 200	O_2_	10	E	14	57	Si	45 nm
[29]	Cotton textile	RF, 30	MA	20	D	74	25		SEM
